# The potential value of LC-MS non-targeted metabonomics in the diagnosis of follicular thyroid carcinoma

**DOI:** 10.3389/fonc.2022.1076548

**Published:** 2022-12-22

**Authors:** Jiali Qin, Yang Yang, Wei Du, Gang Li, Yao Wu, Ruihua Luo, Shanting Liu, Jie Fan

**Affiliations:** ^1^ Department of Head Neck and Thyroid Surgery, Affiliated Cancer Hospital of Zhengzhou University, Henan Cancer Hospital, Zhengzhou, Henan, China; ^2^ Department of Nephrology, The First Affiliated Hospital of Zhengzhou University, Zhengzhou, Henan, China; ^3^ Department of Anatomy, Zhengzhou University, Zhengzhou, Henan, China

**Keywords:** follicular thyroid carcinoma, metabonomics, lipid metabolites, RAS, LysoPA

## Abstract

**Background:**

To explore the metabolic differences of follicular thyroid carcinoma (FTC) by metabonomics, to find potential biomarkers for the diagnosis of FTC, and to explore the pathogenesis and diagnosis and treatment strategies of FTC.

**Method:**

The metabonomics of 15 patients with FTC and 15 patients with follicular thyroid nodules(FTN) treated in Henan Cancer Hospital were analyzed by liquid chromatography-mass spectrometry (LC-MS).

**Results:**

The analysis showed that the metabolite profiles of FTC tissues could be well distinguished from those of control tissues, and 6 kinds of lipids were identified respectively, including lysophosphatidic acid(LysoPA) [LysoPA(0:0/18:0),LysoPA(0:0/18:2(9Z,12Z)],LysoPA[20:4(8Z,11Z,14Z,17Z)/0:0)]; phosphatidic acid(PA) [PA(20:3(8Z,11Z,14Z)/0:0),PA(20:4(5Z,8Z,11Z,14Z)/0:0),PA(20:5(5Z,8Z,11Z,14Z,17Z)/0:0)]; lysophosphatidylcholine(LPC) [LPC(18:1),LPC(16:0),LPC[16:1(9Z)/0:0],LPC(17:0),LPC[22:4(7Z,10Z,13Z,16Z),LPC(20:2(11Z,14Z); phosphatidylcholine(PC)(PC(14:0/0:0),PC(16:0/0:0); sphingomyelin(SM) (d18:0/12:0); fatty acid(FA)(18:1(OH3)]. There are 2 kinds of amino acids, including L-glutamate,L-glutamine.There are 3 other metabolites, including retinol,flavin adenine dinucleotide,androsterone glucuronide.Lipid metabolites are the main metabolites in these metabolites.The metabolic pathways related to FTC were analyzed by KEGG and HMDB, and 9 metabolic pathways were found, including 4 amino acid related metabolic pathways, 1 lipid metabolic pathways and 4 other related pathways.

**Conclusion:**

There are significant differences in many metabonomic characteristics between FTC and FTN, suggesting that these metabolites can be used as potential biomarkers. Further study found that LysoPA and its analogues can be used as biomarkers in the early diagnosis of FTC.It may be related to the abnormal metabolism of phospholipase D (PLD), the key enzyme of LysoPA synthesis caused by RAS pathway. At the same time, it was found that the metabolic pathway of amino acids and lipids was the main metabolic pathway of FTC. The abnormality of LysoPA may be the cause of follicular tumor carcinogenesis caused by lipid metabolic pathway.

## 1 Introduction

Follicular thyroid tumor mainly include FTC, atypical follicular thyroid adenoma and follicular thyroid adenoma(FTA). FTC is one of the highly differentiated malignant tumors, and its incidence is second only to papillary thyroid carcinoma (PTC). The main reason for its occurrence is the abnormal differentiation of thyroid follicular epithelial cells (1). In 2017, WHO divided FTC into three types: slightly invasive type (only invading the capsule), intracapsular vascular infiltrating type and extensive infiltrating type. Lymph node metastasis in FTC was less common than that in PTC, but distant tissue or organ metastasis was easy to occur (2). At present, there are few studies on the pathogenesis of FTC, and the clinical diagnosis and treatment of FTC are mainly focused on imaging examination, fine needle aspiration cytology and so on.Fine needle aspiration cytology(FNAC) is currently the most accurate method to evaluate the benign and malignant thyroid nodules, but there are still 20% to 30% follicular tumors that cannot be determined by FNAC, and FTC is easily confused with FTA in clinical diagnosis, resulting in misdiagnosis (3). Clinically, there is an urgent need for a highly sensitive, specific, efficient, non-invasive and widely used objective index for the diagnosis of thyroid nodules. Therefore, it is necessary to find a stable and reliable tumor molecular marker to assist the diagnosis of FTC.

Metabolomics is a subject of qualitative and quantitative analysis of low molecular weight metabolites in an organism or cell to monitor the changes of chemical products in living cells. It has significant advantages in the early screening of tumor markers (4). The most commonly used analytical methods in metabonomics are nuclear magnetic resonance and liquid or gas chromatography-tandem mass spectrometry(LC/GC-MS) (5). At present, the application of metabonomics in tumor diagnosis is mainly focused on gastric cancer (6), liver cancer (7), lung cancer (8), breast cancer (9), prostate cancer (10) and so on. Metabonomic studies related to thyroid cancer are mainly focused on thyroid papillary carcinoma.Skorupa (11) analyzed 38 cases of PTC, 32 benign thyroid nodules (BTNs) and 112 non-tumor tissue samples by high-resolution magic angle rotation nuclear magnetic resonance technique. It was found that the levels of alanine and lysine in PTC tissue samples were higher than those in non-tumor lesions, while sphingositol content increased in BTNs. In addition, Aboosb R (12) analyzed thyroid nodule patients (including 19 patients with PTC and 16 patients with nodular goiter) and 20 healthy controls by GC-MS. It was found that there were differences in amino acid metabolism, tricarboxylic acid cycle, fatty acid, purine and pyrimidine metabolism between the two groups. At present, few papers on metabonomics related to FTC have been published. In this study, Liquid chromatography-tandem mass spectrometry (LC-MS) metabonomics was used to detect FTC and FTN tissue samples, to screen differential metabolites, to find abnormal metabolic pathways, to explore the potential biomarkers and pathogenesis of FTC, and to provide basis for early diagnosis and treatment of FTC.

## 2 Materials and methods

### 2.1 Study subjects

The subjects were from the patients treated in Henan Cancer Hospital. All the patients in the experimental group were confirmed to be FTC by operation and pathological examination.The experimental group will meet the following items: a). It was confirmed by pathology as FTC;b). No history of other cancers; c). Age ≥ 18 years old; d). No history of blood transfusion; e). No history of taking immunosuppressive drugs. The control group was matched with case frequency according to age and sex. The study was carried out with the informed consent of the subjects.

### 2.2 Sample preparation

Take 50mg solid sample or 100 μl liquid sample into 1.5ml centrifuge tube, add 400 μ l extract (acetonitrile: methanol = 1:1), after vortex mixing for 30s, extract 30min (5 °C, 40KHz) by low temperature ultrasonic extraction, place the sample at-20°C, 30 min, 4°C, 13000g centrifugal 15min, remove the supernatant, dry with nitrogen, re-dissolve 120 μ l complex solution (acetonitrile: water = 1:1), and extract 5min (5 °C,40KHz), 4°C, 13000g centrifugal 5min, the supernatant was removed to the injection vial with internal intubation for analysis.

### 2.3 QC samples LC-MS analysis

All the sample metabolites of the same volume were mixed into quality control samples (QC). In the process of instrumental analysis, one QC sample was inserted into every 10 samples to investigate the repeatability of the whole analysis process.

### 2.4 LC-MS analysis

The instrument platform of this LC-MS analysis is AB SCIEX’s UPLC-TripleTOF system of ultra high performance liquid chromatography tandem time of flight mass spectrometry. Chromatographic conditions: 10ul samples were separated by BEH C18 column (100mm × 2.1 mm I.D., 1.8 μ m) and then detected by mass spectrometry. Mobile phase A: water (containing 0.1% formic acid), mobile phase B: acetonitrile/isopropanol (1pm 1) (containing 0.1% formic acid). Separation gradient: 0-3 min, mobile phase A from linear 95% to 80%, mobile phase B from linear 5% to 20% min, mobile phase A from linear 80% to 5%, mobile phase B linear from 20% to 95% min, mobile phase A linear to 5%, mobile phase B linear to 95%. 13.0-13.1 min, the linearity of mobile phase An increases from 5% to 95%, the linearity of mobile phase B decreases from 95% to 5%, the linearity of mobile phase A maintains 95%, and the linearity of mobile phase B maintains 5%. The flow rate is 0.40 mL/min and the column temperature is 40 °C. Mass spectrometry conditions: the sample mass spectrometry signal was collected in positive and negative ion scanning mode, and the mass scanning range (m/z) was 50-1000. Ion spray voltage, positive ion voltage 5000V, negative ion voltage 4000V, de-cluster voltage 80V, fog 50psi, auxiliary heater 50psi, air curtain gas 30psi, ion source heating temperature 500°C, 20-60V cycle collision energy.

### 2.5 Data preprocessing and database search

After the completion of the computer, the LC-MS raw data are imported into the metabonomics processing software Progenesis QI (Waters Corporation, Milford, USA) for baseline filtering, peak identification, integration, retention time correction and peak alignment, and finally a data matrix of retention time, mass-to-charge ratio and peak intensity is obtained. The data matrix uses the 80% rule to remove the missing values, that is, to retain at least one group of samples with non-zero values of more than 80%. Then fill the vacancy value (the minimum value in the original matrix). In order to reduce the error caused by sample preparation and instrument instability, the response intensity of the essential spectrum peak of the sample is normalized by the sum normalization method, and the normalized data matrix is obtained.At the same time, the variables with relative standard deviation (RSD) > 30% of QC samples are deleted and logarithmized by log10 to get the final data matrix for follow-up analyses. At the same time, the metabolite information was obtained by matching the mass spectrometry information of MS and MSMS with the metabolic public database HMDB (http://www.hmdb.ca/) and Metlin (https://metlin.scripps.edu/) database. Metabolite identifications were accepted if they could be established on a basis of at least one unique metabolite identified with a high confidence (FDR < 1%). Metabolite abundances were calculated using intensity of all precursors. For each case, normalized abundance by SEQUEST searches for the metabolite. The metabonomics data of a specimen was determined by the metabolite with the largest normalized abundance.

### 2.6 Analysis of differential metabolites

The preprocessed data is uploaded to Meiji biological cloud platform (https://cloud.majorbio.com) for data analysis. The R software package ropls (Version1.6.2) carries out orthogonal least square discriminant analysis (OPLS-DA), and uses 7 cycles of interactive verification to evaluate the stability of the model. In addition, student’s t test and multiple of difference analysis were performed. The selection of significant differential metabolites was based on the variable weight value (VIP) obtained by OPLS-DA model and the p value of student’s t test. The metabolites with VIP > 1 and p <0.05 (student’s t test) were significant differential metabolites. A total of 11 differential metabolites were screened.

### 2.7 Bioinformatics analysis

Notes for KEGG access: the metabolic pathways were annotated by KEGG database (https://www.kegg.jp/kegg/pathway.html) to obtain the pathways involved by differential metabolites. KEGG enrichment analysis: the pathway enrichment analysis was carried out by Python software package scipy.stats, and the biological pathway most related to the experimental treatment was obtained by Fisher accurate test. HMDB compound classification: the differential identified metabolites were classified by HMDB compound classification database. IPath metabolic pathway analysis: iPath3.0 (http://pathways.embl.de) was used to visually analyze the metabolic pathways involved in metabolic sets to view the metabolic pathway information of the whole biological system.

## 3 Results

### 3.1 Metabolism of FTC and FTN

**Figure 1:**
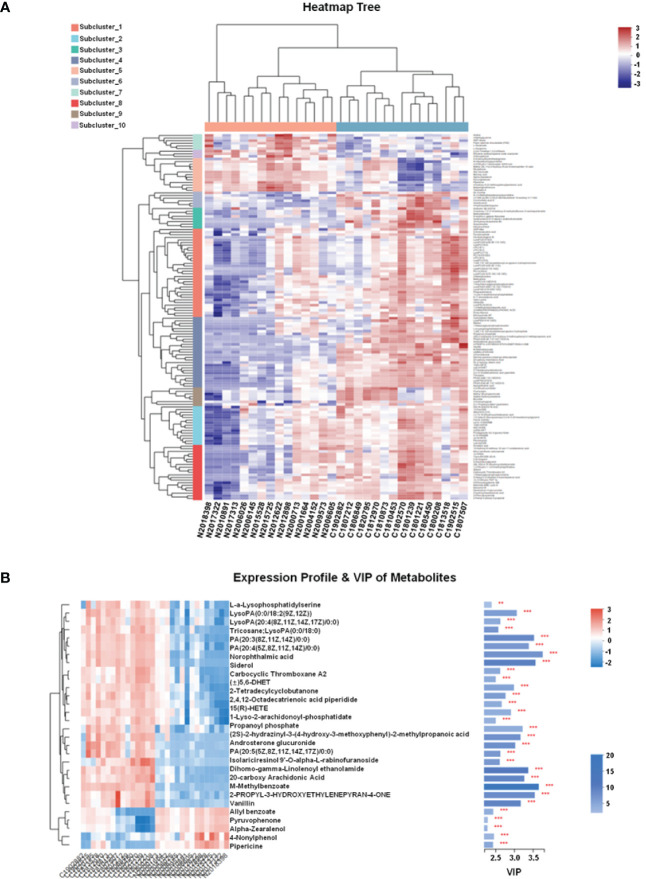
FTC and FTN tissue metabolites clustering heat map and VIP diagram. **(A)** FTC and FTN tissue metabolites clustering heat map. **(B)** Metabolite expression profile and VIP diagram. Each column in the Figure represents a sample and each row represents a metabolite. The color in the Figure indicates the relative expression of the metabolite in this group of samples.There is a tree of metabolites clustering on the left and the names of metabolites on the right. The closer the two metabolites branch to each other, the closer their expression is.The tree view of the sample clustering at the top and the name of the sample at the bottom. In the VIP diagram,on the right side is the metabolite VIP bar chart, the bar length represents the contribution of the metabolite to the difference between the two groups, the default is not less than 1, the higher the value, the greater the difference between the two groups.The bar color indicates that there is a significant difference in metabolites between the two groups, that is, the smaller the P_value, the larger the-log10 (P-value) and the darker the color. On the right, **P < 0.01, and ***represents P < 0.001.

**Figure 2:**
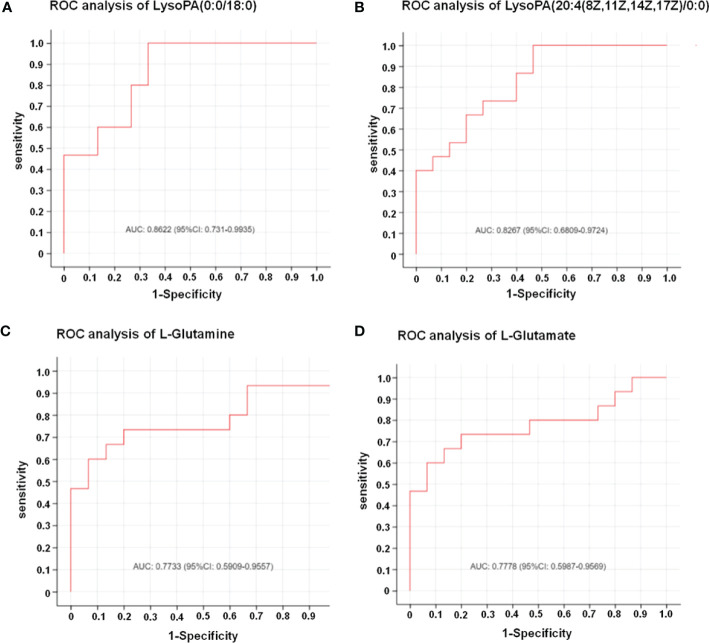
The results of ROC analysis showed that the four metabolites had significant diagnostic value for FTC. **(A)** Result of ROC analysis of LysoPA(0:0/18:0). AUC: 0.8622 (95%CI: 0.731-0.9935). **(B)** Result of ROC analysis of LysoPA(20:4(8Z,11Z,14Z,17Z)/0:0)). AUC: 0.8267 (95%CI: 0.6809-0.9724). **(C)** Result of ROC analysis of L-glutamine. AUC: 0.7733 (95%CI: 0.5909-0.9557). **(D)** Result of ROC analysis of L-glutamate. AUC: 0.7778 (95%CI: 0.5987-0.9569). The X axis in the picture is 1-Specificity; Y axis is Sensitivity;The AUC marked in the Figure is the area under the corresponding curve;AUC values are usually between 0.5 and 1.0;When AUC > 0.5, the closer the AUC is to 1, the better the diagnostic effect is;The accuracy of AUC is lower at 0.5-0.7, that of AUC is higher when 0.7-0.9, and that of AUC above 0.9 is extremely high.

**Figure 3:**
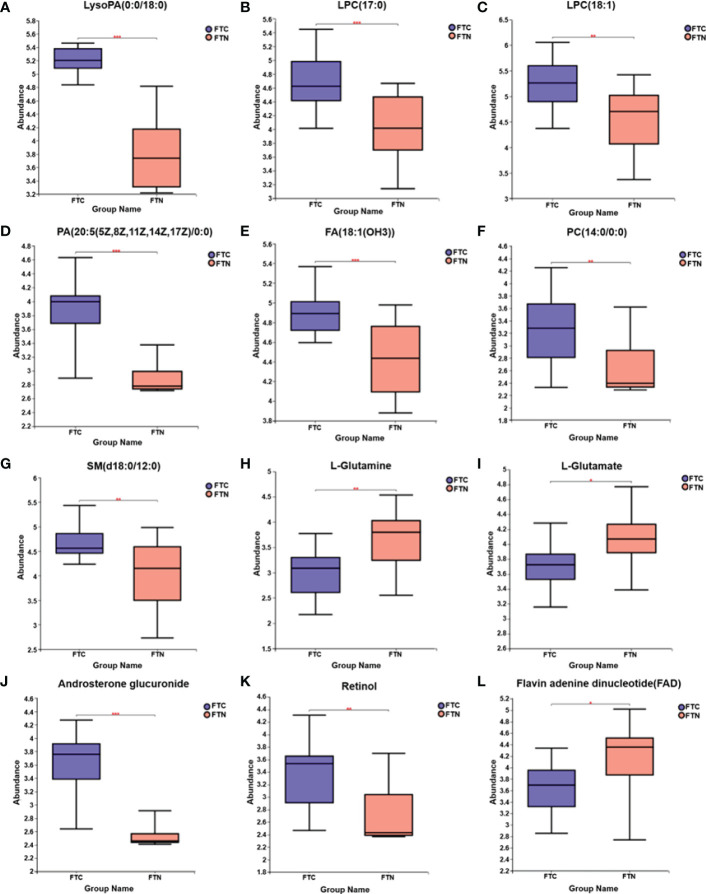
Box diagram of medium metabolite distribution for each group of samples. **(A-L)** Box diagrams of LysoPA(0:0/18:0), LPC(17:0), (LPC(18:1), PA(20:5(5Z,8Z,11Z,14Z,17Z)/0:0), FA(18:1(OH3)), PC(14:0/0:0), SM(d18:0/12:0), L-glutamine, L-glutamate, androsterone glucuronide, retinol, flavin adenine dinucleotide distributions in FTC and FTN groups, respectively. The line in the middle of the box represents the median relative abundance of metabolites.The upper and lower bottom of the box are the upper quartile (Q3) and the lower quartile (Q1) of the relative strength of metabolites, respectively.The height of the box reflects the degree of fluctuation of the data to some extent.The upper and lower edges represent the maximum and minimum values of the set of data.The data outside the box can be understood as “outliers” in the data.* represents P < 0.05,** represents P < 0.01, and *** represents P < 0.001.

In this study, LC-MS was used to analyze FTC tissue and FTN tissue control group. A total of 140 metabolites, including lipids, amino acids, carbohydrates, organic acids and esters, were identified and quantified. Cluster heat map analysis of differential metabolites between FTC and control groups showed the differences of 140 metabolites between FTC and FTN groups, and the relative changes of metabolites concentration in different groups (**Figure 1**). According to the ROC curve (**Figure 2**), the metabolic spectrum of thyroid tissue shows representative metabolites: lipids, amino acids. A total of 11 metabolites were identified, including 6 kinds of lipids, 2 kinds of amino acids and 3 kinds of other metabolites (**Table 1**). The expression of each metabolite is shown in the box diagram (**Figure 3**).

**Table 1:** Differences of characteristic metabolites between experimental group and control group.

Ion mode	Metabolite	VIP_OPLS-DA	VIP_PLS-DA	FC(FTC/FTN)	P	AUC
Neg	LysoPA(0:0/18:0)	3.524	2.948	1.359	6.61E-10	0.862
Neg	LysoPA(0:0/18:2(9Z,12Z))	3.064	2.665	1.331	2.44E-07	0.831
POS	LysoPA(20:4(8Z,11Z,14Z,17Z)/0:0)	2.623	2.304	1.312	4.79E-06	0.827
POS	LPC(18:1)	1.837	1.686	1.155	0.002	0.804
Neg	LPC(16:0)	1.705	1.505	1.120	0.002	0.8
POS	LPC(16:1(9Z)/0:0)	1.467	1.482	1.140	0.014	0.724
Neg	LPC(17:0)	1.982	1.769	1.163	0.0004	0.844
Neg	LPC(22:4(7Z,10Z,13Z,16Z))	1.670	1.502	1.199	0.006	0.773
Neg	LPC(20:2(11Z,14Z))	1.674	1.533	1.208	0.004	0.809
POS	PA(20:3(8Z,11Z,14Z)/0:0)	3.385	2.944	1.472	6.58E-08	0.796
POS	PA(20:4(5Z,8Z,11Z,14Z)/0:0)	3.758	3.284	1.591	1.32E-08	0.782
POS	PA(20:5(5Z,8Z,11Z,14Z,17Z)/0:0)	2.623	2.353	1.333	6.61E-08	0.756
POS	PC(14:0/0:0)	1.684	1.488	1.228	0.003	0.7
POS	PC(16:0/0:0)	1.568	1.436	1.105	0.004	0.687
Neg	FA(18:1(OH3))	1.699	1.524	1.106	0.0004	0.722
POS	SM(d18:0/12:0)	1.743	1.658	1.166	0.005	0.751
Neg	L-Glutamate	1.220	1.191	0.920	0.017	0.778
Neg	L-Glutamine	1.897	1.812	0.821	0.003	0.773
POS	Retinol	1.781	1.593	1.238	0.002	0.722
Neg	Androsterone glucuronide	3.021	2.776	1.422	5.06E-09	0.773
Neg	Flavin adenine dinucleotide	1.479	1.725	0.872	0.01	0.751

(1) Ion mode: the mode of substance detection by mass spectrometer, which mainly includes pos (positive ion mode) and neg (negative ion mode); (2) Metabolite: the name of the metabolite identified; (3) VIP_OPLS-DA: the VIP value of this metabolite in the OPLS-DA model between two groups; (4) FC (Y/ X): the differential expression multiple of the metabolite between the two groups.X, the expression of this metabolite in the control group X; Y, the expression of this metabolite in experimental group Y; X as control; (5) P-value: the result of significance test of the difference of this metabolite between two samples.

### 3.2 Comparison of metabolites between FTC and FTN

#### 3.2.1 Orthogonal partial least-squares discriminant analysisis used to distinguish between FTC and FTN

**Figure 4:**
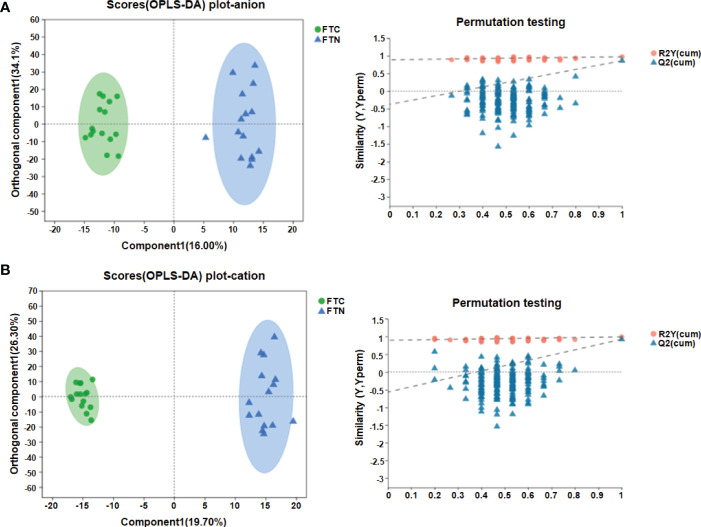
OPLS-DA results showed the efficient discriminant of the model. **(A)** OPLS-DA score chart and permutation test(anion mode); **(B)** OPLS-DA score chart and permutation test(cationic mode). The OPLS-DA score chart is often used to directly show the classification effect of the model. In OPLS-DA score chart, the abscissa is the interpretation degree of Comp1’s first predicted principal component, and the ordinate is the interpretation degree of orthogonal Comp1’s first orthogonal component.In OPLS-DA permutation test, Abscissa represents the permutation retention of permutation test, ordinate indicates the value of R2 (red dot) and Q2 (blue triangle) permutation test, and the two dotted lines represent the regression lines of R2 and Q2 respectively.

In addition, OPLS-DA is carried out, and the OPLS-DA score map filters out the information that has nothing to do with the grouping through orthogonal rotation, so as to better distinguish the differences between groups and improve the efficiency of the model. In the anion mode: the OPLS-DA score map (**Figure 4A**) shows that there are significant differences in metabolites between the control group and the experimental group, which can be well distinguished. OPLS-DA permutation test (**Figure 4A**) (R2Y=0.976 and Q2 = 0.863) shows that these models are reliable. In the cation mode: the OPLS-DA score chart (**Figure 4B**) shows that there are significant differences in metabolites between the control group and the experimental group, which can also be well distinguished.OPLS-DA permutation test (R2Y=0.991 and Q2 = 0.923) shows that these models are reliable (**Figure 4B**).

#### 3.1.2 VIP diagram analysis is used to distinguish between FTC and FTN

Predictive variable importance (VIP) scores based on the OPLS-DA model indicate the potential metabolites as biomarkers (**Figure 1B**). Variables with VIP scores greater than 1.5 are considered important to the classification model. The VIP scores of various metabolites were more than 1.5, including LysoPA, PA and so on.

#### 3.1.3 Volcanogram analysis is used to distinguish FTC from FTN

**Figure 5:**
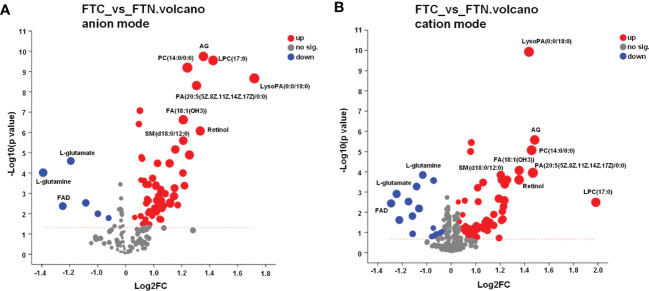
Volcano diagram of metabolite in FTC and FTN. **(A)** Volcano diagram of metabolites identified between FTC and FTN in anion mode. **(B)** Volcano diagram of metabolites identified between FTC and FTN in cation mode. Abscissa is the multiple change value of metabolite expression difference between the two groups, namely log2FC, ordinate is the statistical test value of metabolite expression difference, namely-log10 (p_value) value, the higher the value is, the more significant the difference is, and the values of horizontal and vertical coordinates are logarithmized. Each point in the Figure represents a specific metabolite, and the size of the point represents the VIP value. The point on the left is the metabolite of differential down-regulation, and the point on the right is the metabolite of differential up-regulation. The more close to the left and right side and the above point, the more significant the expression difference.

The point on the right side of the volcano chart indicates that the metabolite is up-regulated and the point on the left side indicates that the metabolite is down-regulated. In the anion model of metabolites, most of the differential metabolites in FTC and FTN groups showed an up-regulation trend, while a few metabolites showed a down-regulation trend (**Figure 5A**). In the metabolite cation model, most of the differential metabolites in FTC and FTN groups showed an up-regulation trend, while some metabolites showed a down-regulation trend (**Figure 5B**).

### 3.3 Metabolic pathways affecting FTC

**Figure 6:**
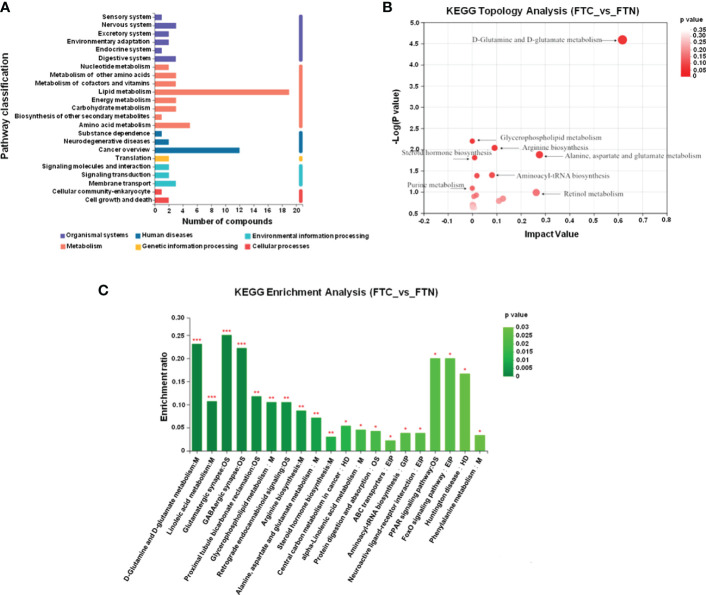
Metabolic pathways involved in FTC. **(A)** KEGG pathway analysis shows that abnormal lipid metabolism pathway plays a key role in the formation of FTC. The ordinate is the secondary classification of the KEGG metabolic pathway, and the Abscissa is the number of metabolites annotated to the pathway. KEGG metabolic pathways can be divided into seven categories: Metabolism,Genetic Information Processing,Environmental Information Processing,Cellular Processes,Organismal Systems,Human Disease and Drug Development. **(B)** KEGG Topology Analysis Bubble Diagram. Note: each bubble in the Figure represents a KEGG Pathway pathway; the horizontal axis represents the relative importance of metabolites in the pathway Impact Value; the vertical axis represents the significant enrichment significance of metabolites in the pathway-log10 (Pvalue); the bubble size represents the Impact Value value; the larger the bubble, the greater the importance of the pathway. **(C)** KEGG enrichment analysis(FTC_vs_FTN). Note: Abscissa denotes pathway name, and ordinate denotes enrichment rate, indicating the ratio of the metabolite number enriched in the pathway to the number of background number to pathway. The higher the ratio, the greater the degree of enrichment.The color gradient of the column indicates the significance of enrichment. The darker the default color, the more significant the enrichment of the KEGG term. Pvalue or FDR < 0.001 is marked as ***, Pvalue or FDR < 0.01 is marked as **, Pvalue or FDR < 0.05 is marked as *.

The metabolic pathways related to FTC and reliable results were analyzed by Kyoto Encyclopedia of Genes and Genomes(KEGG) and human metabolome database(HMDB), and the KEGG metabolic pathways could be divided into six categories (**Figure 6A**): metabolism, genetic information processing,environmental information processing,cellular processes,organismal systems and human diseases. The results of pathway analysis are shown in **Table 2** and **Figure 6B**, and seven meaningful pathways have been found, including D-glutamine and D-glutamate metabolism, alanine, aspartate and glutamate metabolism, arginine biosynthesis, glycerophospholipid metabolism, glyoxylate and dicarboxylate metabolism, aminoacyl-tRNA biosynthesis and steroid hormone biosynthesis, which were significantly correlated with FTC. In addition, in order to expand the understanding of the metabolic pathways related to FTC, the enrichment analysis module of the metabolic analysis system (**Figure 6C**) was used to find a number of pathways significantly related to FTC, including two metabolic pathways, including linoleic acid metabolism and glutathione metabolism.

**Table 2:** Pathway analysis of metabolic changes in FTC.

Pathway Desciption	Total	Impact	P_value
D-Glutamine and D-glutamate metabolism	2	0.618	2.58E-05
Alanine, aspartate and glutamate metabolism	2	0.276	0.013
Arginine biosynthesis	2	0.092	0.009
Steroid hormone biosynthesis	2	0.010	0.016
Glycerophospholipid metabolism	10	0.009	0.006
Purine metabolism	2	0	0.082
Aminoacyl-tRNA biosynthesis	2	0.081	0.040
Retinol metabolism	1	0.263	0.103
Glyoxylate and dicarboxylate metabolism	2	0.019	0.041

Note: (1) Pathway Description is the name of the channel; (2) Total: the number of metabolites identified in the pathway; (3) Impact: comprehensive importance score of the path, with a total score of 1; Calculated according to the relative position of metabolites in the pathway; (4) P-value: the result of significance test of the difference of this metabolite between two samples.

## 4 Discussion

Thyroid cancer(TC) is one of the malignant tumors with the fastest increasing incidence in the world in recent years (13). With the improvement of living standards, routine physical examination is becoming more and more popular. About 1/5 of adults find thyroid nodules in the physical examination (14). At present, FNAB is the gold standard for the diagnosis of thyroid cancer, but due to insufficient sampling and the difficulty in determining the nature of follicular thyroid tumors, 20% to 30% of thyroid nodules cannot be identified clinically.Of the thyroid nodules of uncertain nature, 10% to 30% were diagnosed as malignant after operation (15, 16). Therefore, it is necessary to have a highly sensitive, specific, efficient, non-invasive and widely used objective index for the diagnosis of thyroid nodules. Metabonomics is a discipline that comprehensively and systematically describes all small molecular metabolites in biological samples. It has obvious advantages in disease diagnosis and research (17, 18).

The main methods of metabolic research include nuclear magnetic resonance (NMR) spectroscopy and mass spectrometry (MS), gas chromatography (GC) and liquid chromatography (LC) and other separation techniques. Nuclear magnetic resonance technology has the advantages of non-selective and non-destructive analysis of samples, low sample consumption, simple sample pretreatment, non-invasive and other advantages, and can be used for the analysis of various body fluids (19). However, its sensitivity and resolution need to be improved, and the required sample concentration is high, so it is impossible to analyze the components with large concentration difference at the same time. Mass spectrometry has wide adaptability, high specificity and sensitivity, but its disadvantage lies in low selectivity and poor ability to identify a large number of spectral peaks. The chromatographic technology has strong separation ability and accurate quantitative analysis, but the ability of qualitative analysis is weak. Liquid chromatography-mass spectrometry can make up for their shortcomings, with good repeatability, high sensitivity, strong qualitative and quantitative ability, and can detect most of the organic molecules in biological samples (20). In this study, 15 cases of FTC and 15 cases of FTN were analyzed by liquid chromatography-mass spectrometry (LC-MS). It was found that there were significant differences in lipids and amino acids between FTC and control tissues. It can be used in the diagnosis of FTC.

The development of thyroid cancer requires the existence of specific conditions under which cell metabolism is reprogrammed to meet its bioenergy and biosynthesis needs and to allow cells to proliferate uncontrolled. Therefore, the low molecular weight metabolites of cancer tissues are significantly different from those of other tissues. Metabonomics technology can accurately detect the differences between these metabolites and find the relative relationship between these metabolites and physiological and pathological changes to diagnose diseases and monitor health. As an important metabolite, lipids participate in the self-assembly of phospholipids to form biofilms and participate in cell differentiation and signal transduction as second messengers. Previous studies (21) have found that the occurrence and development of tumors are closely related to the changes of lipid levels. In this study, our experimental results showed that the level of PA, SM in FTC was significantly increased, and the AUG value was more than 0.7. This may be the capsular infiltration or vascular infiltration of FTC, and the tumor tissue penetrates the capsule, resulting in changes in the composition of the cell membrane. It causes abnormal levels of phospholipids and metabolites related to cell membrane synthesis in the body.

Guo (22) studied the lipid changes of TC and BTN tissue and blood by tissue mass spectrometry and serum lipid spectrum analysis. It was found that the biomarker group composed of phosphatidic acid (PA) (36:3) and sphingomyelin (SM) (34:1) could distinguish between benign and malignant tumors, with an AUC value of 0.961, a sensitivity of 87.8% and a specificity of 92.9%, which was similar to our results. In addition, our study also found that the level of FA, PC is higher than that of FTN, which may be related to the increase of thyrotropin and the corresponding increase of thyroxine synthesis in patients with FTC, resulting in abnormal plasma lipid levels.On the other hand, there are great differences in lipid metabolism between benign and malignant nodules. Circulating free fatty acids are important for energy replenishment, especially when glucose is insufficient. Excessive proliferation of malignant tumor cells, insufficient energy supply of glucose oxidation, energy supply by fat oxidation, which also indirectly leads to abnormal lipids. Guo (23) performed lipid imaging and analysis on tissue samples from six different types of cancer, including 124 cases of TC and 122 controls (healthy subjects and BTN samples). The results showed that the levels of 10 lipids in TC serum changed, including 3 phosphatidylcholines, 6 phosphatidic acids and 1 sphingomyelin, which indicated that there were differences in lipid metabolism between TC and other thyroid tissues.

Interestingly, we found that a variety of lysophosphatidic acid (LysoPA) levels are generally increased in FTC, and LysoPA is produced under physiological and pathophysiological conditions of cells and extracellular fluid. It mediates a variety of cellular responses and activities, including cell proliferation, migration, invasion, cytokine production, reactive oxygen species (ROS) production and cancer cell progression (24). The latest study (25) found that LysoPA can act on specific G protein-coupled receptors and regulate many metabolic processes, such as vascular development, immunity and carcinogenesis. In addition, LysoPA has been found to induce the characteristics of many cancers, including cellular processes such as proliferation, survival, migration, invasion and neovascularization. So far, LysoPA has been detected in many human body fluids, including plasma (26), serum, cerebrospinal fluid (27), pleural effusion (28) and so on.

LysoPA has two main biosynthetic pathways. First, phosphatidic acid (PA) is formed by the decomposition of phospholipids by phospholipase D (PLD), and then converted by phospholipase A1 or A2 (PLA1 and PLA2) to LysoPA. Second, lysophospholipids are produced by the corresponding phospholipids through PLA1 or PLA2. They are then cut into LysoPA by the action of lysophospholipase D (also known as autophagotoxin (ATX)) (25). Related studies have found that abnormal LysoPA levels are associated with a variety of diseases, including breast cancer (29), ovarian cancer (30) and so on. At present, the source and mechanism of the increase of LysoPA in FTC are not clear. We guess: 1. LysoPA can recognize two kinds of G protein coupled receptors: LysoPA1-3 receptor and LysoPA4-6 receptor, which belong to endothelial gene (EDG) family and non-endothelial gene family respectively.Some studies (31) found that the expression of EDG4 receptor mRNA in FTC increased by 3 times, and had a high affinity for LysoPA, which led to the increase of LysoPA. 2.RAS pathway is the key pathway of FTC (32), and phospholipase D (PLD) is the key enzyme to produce LysoPA. We speculate that the metabolism of RAS protein in RAS pathway is abnormal during follicular thyroid tumor carcinogenesis. It has been found that Ras protein is a key element in the regulation of phospholipase D (PLD) (33). Therefore, the increase of LysoPA in FTC may be due to the abnormal metabolism of Ras protein, which leads to the increase of phospholipase D synthesis and further leads to the increase of LysoPA.3. Lysophospholipids are produced by the corresponding phospholipids through PLA1 or PLA2, and then cut into LysoPA by lysophospholipase D (also known as autophagy toxin (ATX)). The resulting LysoPA is dephosphorylated by phospholipid phosphatase (LPP) and degraded to monoacylglycerol (MAG), or converted to PA by acyltransferase. The increase of LysoPA in FTC may be the result of ATX-LysoPA-LPP axis misalignment. 4. FTC can be diagnosed when follicular thyroid tumor invades blood vessels, capsule and surrounding tissue.LysoPA acts on tissues to produce endothelin and angiogenic factors (vascular endothelial growth factor (VEGF), interleukin (IL)-6, etc.), which can be used as paracrine growth factors of malignant cells. The increase of LysoPA in FTC may be caused by tumor invasion of surrounding blood vessels and tissues. These results suggest that the different expression of LysoPA in different tissues may provide a non-invasive, simple and accurate method for the diagnosis of FTC.

The characteristics of low molecular weight metabolites in tumor tissues are significantly different from those in other tissues. These metabolite characteristics can be expressed in two ways:1. The concentration of low molecular weight metabolites is constantly changing at each stage of tumor tissue progression, and these differential metabolites can be detected by metabonomics to diagnose diseases. 2. There are also many changes in metabolic pathways involved in each stage of tumor tissue progression. After annotating KEGG and HMDB and analyzing their pathways and enrichment, we found that important metabolic pathways are related to FTC. The changes of amino acids in FTC mainly include L-glutamate and L-glutamine. The KEGG pathways involved in L-glutamate and L-glutamine include D-glutamine and D-glutamate metabolism, alanine, aspartic acid and glutamate metabolism, arginine biosynthesis, glutathione metabolism and so on. Recently, Gu (34) studied 33 patients with TC and 137 healthy controls by amino acid analyzer. It was found that the levels of threonine and arginine in TC samples were higher, while those of aspartic acid, glutamic acid and proline were lower. Shen (35) and other researchers identified 31 metabolites by comparing the sera of 37 patients with distant metastasis of TC with those of 40 patients with BTN. They are related to glucose, amino acids, lipids, vitamins metabolism and diet/intestinal microbiota interaction. Pathway analysis shows that alanine, aspartic acid and glutamate metabolism and inositol phosphate metabolism are the most related pathways. These findings all support our results. Therefore, the increase of L-glutamate and L-glutamine in thyroid tissue should be noticed, as they may be new tumor markers of FTC. At present, the mechanism of changes in amino acid metabolic pathways related to FTC is still unclear, but it has been found that there are many related metabolic pathways, with the deepening of research, more amino acid metabolic pathways may be found. Another type of KEGG pathway we found is related to lipids, which is glycerol phospholipid metabolic pathway, which involves metabolites such as PC, PA, SM, FA, LysoPA and so on. Miccoli (36) analyzed 28 cases of thyroid papillary carcinoma, 40 cases of thyroid follicular lesions and 4 cases of benign nodules by high resolution magic angle rotation NMR. It was found that benign and malignant tumors could be distinguished by metabonomics. Choline and lipid metabolism were abnormal in malignant samples (thyroid papillary carcinoma and FTC). This is similar to our findings, and we speculate that abnormal LysoPA may be the cause of follicular tumor carcinogenesis caused by lipid metabolic pathway. In addition, KEGG pathways involved in FTC also include Glyoxylate and dicarboxylate metabolism, Aminoacyl-tRNA biosynthesis, Steroid hormone biosynthesis,Linoleic acid metabolism. There are few studies on these pathways related to thyroid cancer, and a large number of sample studies are still needed to confirm their exact relationship.

## Conclusion

In short, there are significant differences in a variety of metabonomic characteristics between FTC and FTN, suggesting that these metabolites can be used as potential biomarkers. At the same time, our study found that LysoPA has a very strong diagnostic ability for FTC, which may be related to the abnormal metabolism of phospholipase D (PLD), the key enzyme of LysoPA synthesis caused by RAS pathway. In addition, LysoPA can be detected in a variety of human body fluids, which has the potential to be used in clinic, but it still needs a large number of experiments to confirm. We also found that Amino acid metabolic pathway and lipid-related metabolic pathway may be the key pathways of follicular tumor carcinogenesis, which need to be further studied to explore its potential mechanism and its role in the development of FTC.This study provides new insights into the diagnosis of FTC by studying the differences of related metabolites and abnormal metabolic pathways of FTC, and explores the potential biomarkers of FTC, which has great potential in the diagnosis and treatment of FTC.

## Data availability statement

The original contributions presented in the study are included in the article/**Supplementary Material**. Further inquiries can be directed to the corresponding author.

## Ethics statement

Our study was approved by the Institutional Ethics Committee of the Henan Cancer Hospital. All participants signed informed consent to medical research before the initial treatment, and all experiments were performed in accordance with the relevant guidelines and regulations.

## Author contributions

JF and JQ: conceptualization and original draft; JF and YY: data curation and methodology; JQ, JF and YY: manuscript review and editing; JF, DW and JQ: software; SL and RL: investigation; YW and GL: project administration; JF, RL and SL: supervision, validation, and visualization. All authors contributed to the article and approved the submitted version.
