# Supplementary figures and images for: The potential value of LC-MS non-targeted metabonomics in the diagnosis of follicular thyroid carcinoma
Source: Front Oncol. 2022 Dec 22;12:1076548. doi: 10.3389/fonc.2022.1076548 (PMC9814718; doi:10.3389/fonc.2022.1076548)

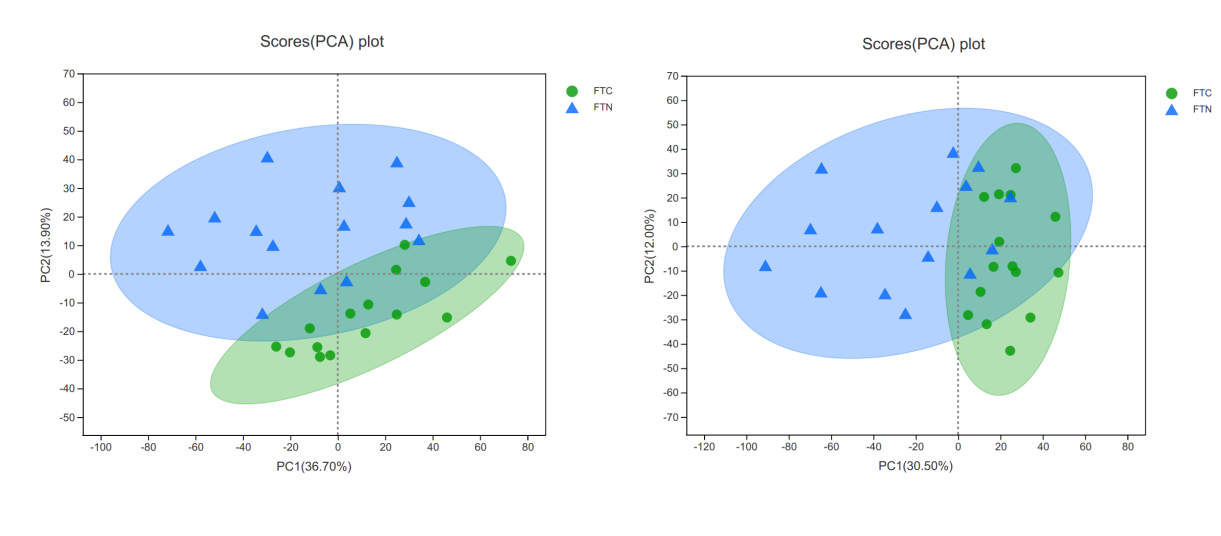

Supplement: Supplementary file 1 [file DataSheet_1.zip › supplementary files/Principal component analysis (Supplementary Data).png]
